# Detection of a second malignancy in prostate cancer patients by using [^18^F]Choline PET/CT: a case series

**DOI:** 10.1186/s40644-016-0085-1

**Published:** 2016-08-31

**Authors:** Martina Sollini, Francesco Pasqualetti, Marzio Perri, Gabriele Coraggio, Paolo Castellucci, Massimo Roncali, Roberto Boni, Elena Lazzeri, Maria Galeandro, Fabiola Paiar, Annibale Versari, Paola Anna Erba

**Affiliations:** 1 Humanitas University, Rozzano, Milan, Italy; 2 Radiation Oncology Unit, Azienda Ospedaliero-Universitaria Pisana, Pisa, Italy; 3 Istituto Radiologico Toscano, Alliance Medical, Pistoia, Italy; 4 Nuclear Medicine Unit, Policlinico S. Orsola-Malpighi, Bologna, Italy; 5 Nuclear Medicine Unit, Arcispedale Santa Maria Nuova, IRCCS - Reggio Emilia, Reggio Emilia, Italy; 6 Regional Center of Nuclear Medicine, University of Pisa, 56125 - Via Roma, 56 Pisa, Italy; 7 Radiation Oncology Unit, Arcispedale Santa Maria Nuova, IRCCS - Reggio Emilia, Reggio Emilia, Italy

**Keywords:** Prostate cancer, [^18^F]Choline PET/CT, Second malignancy, Incidentaloma

## Abstract

**Background:**

The role of radiolabeled choline (Cho) in patients with biochemical recurrence after radical treatment for prostate cancer (PCa) is well established. Its widespread clinical use has prompted the depiction of incidentalomas, unusual sites of metastatic lesions, as well as false positive and negative cases. We reported a series of patients affected by biochemical recurrence of PCa imaged by [^18^F]Cho positron emission tomography/computed tomography (PET/CT) which resulted suspected for a second malignancy.

**Case presentation:**

[^18^F]Cho PET/CT was performed in patients with biochemical PCa recurrence. From an internal clinical database we identified patients in which PET/CT resulted suspected for a second malignancy. A second malignancy was suspected in presence of “unusual” site of [^18^F]Cho uptake not consistent with clinical-instrumental history. Histology was used as reference standard for final diagnosis.

Seven PCa patients (76 years, 71–84 years) with the suspicion of a second tumor based on [^18^F]Cho PET/CT findings were identified. Mean value of PSA at the time of [^18^F]Cho PET/CT was 2,37 ng/mL. The median time between PCa diagnosis and PET/CT was 6 years (range 0–14 years). In two cases history of a second malignancy (lung cancer and cutaneous basocellular carcinoma) was known (diagnosed 12 and 6 years after PCa, respectively). PET/CT identified 13 sites of [^18^F]Cho uptake (lung = 5, lymph node = 7, bone = 1). Final diagnosis was consistent with lung cancer in 5/7 cases (first diagnosis = 4/5, recurrence = 1/5), colorectal cancer and nodal metastases from melanoma in 1 case each.

**Conclusions:**

Although the clinical usefulness of Cho PET/CT for detecting cancer lesions other than prostate origin is known, for those patients who undergo this examination according to indication, the diagnosis of a second tumor has a significant impact on their therapeutic management.

## Background

Prostate cancer (PCa) is a clinically heterogeneous disease characterized by an overall long natural history in comparison with other solid tumors, showing a wide spectrum of biological behavior ranging from indolent to aggressive [1]. Despite highly successful treatments for localized prostate cancer, approximately 15–40 % of patients will experience a detectable rise in the serum prostate specific antigen (PSA) level (i.e. biochemical failure) within 10 years after primary treatment [2]. In patients with biochemical failure, imaging plays a critical role in distinguishing between local recurrence and distant spread of disease when developing appropriate treatment strategies [3]. The biochemical process target of choline-based tracers is the synthesis of cell membrane [4]. The uptake of radiolabeled choline would reflect the proliferative activity by estimating membrane lipid synthesis. Tumor cells with high proliferation rate, will have high uptake of choline to keep up with increased demands for the synthesis of phospholipids [5]. Based on these premises and the need of radiotracer alternative to [^18^F]fluorodeoxyglucose to image some neoplasms, starting from 1970s radiolabeled choline was introduced as positron emission tomography tracer to image brain tumor and PCa [6, 7]. Radiolabeled choline (Cho) positron emission tomography/computed tomography (PET/CT) has been widely investigated as diagnostic tool to restage PCa in case of biochemical recurrence and has been shown to be of value in the detection of local recurrence and distant metastases [8]. It has been shown that in almost 50 % of patients, choline PET/CT findings impact on patient’s management [8, 9]. However, choline PET/CT may also be successfully used to image a variety of other malignancies [8, 10–13]. As for many other tumors, also in PCa, the detection of a second malignancy really change the management of the patient and the treatment strategies [14].

In this paper, we addressed the added value of fluorocholine ([^18^F]Cho) PET/CT in PCa patients to identify a second malignancy underlying the need of a proper expertise in the interpretation of the images.

## Case presentation

We reported in the present series seven PCa patients selected from an internal clinical database, in whom based on [^18^F]Cho PET/CT results, a second malignancy was suspected. All patients were referred to the Nuclear Medicine Unit to perform a fluorocholine PET/CT. All patients presented a biochemical PCa recurrence at the time of [^18^F]Cho PET/CT. The suspicious of a second malignancy was supposed based on PET/CT findings (i.e. “unusual” site of [^18^F]Cho uptake not consistent with clinical-instrumental history) according to previously reported by Calabria et al. [11].

Images were acquired using a dedicated PET/CT tomograph (Discovery, GE or Gemini, Philips). In our experience the prevalence of a diagnosis of a second malignancy detected by [^18^F]Cho PET/CT in PCa patients was 0.53 %. All patients provided informed consent for PET/CT examination and for the use of personal data. Baseline patients characteristics and main results are detailed in Table 1.

**Table 1:** Baseline patients characteristics and main results

Pt	Age, years	Curative PCa treatment(s)	PSA (ng/mL)	PET/CT			Final diagnosis	Treatment	
				Year	Clinical purpose	[^18^F]Fluorocholine uptake (site)		Expected	Undertaken
#1	72	RP (2004) + ADT (ongoing)	0.11	2010	Diagnosis of incidental bone lesion (PCa mts ?)	Lung + bone and soft tissues	NSCLC	CHT (docetaxel)	CHT (platinum-based) + palliative EBRT
#2	71	RP (1998) + salvage EBRT (2009)	0.08	2010	Persistence of increased PSA after salvage EBRT	Lung	NSCLC	ADT	CHT (platinum-based)
#3	84	EBRT (1996)	2.62	2008	Biochemical recurrence	Lung + lymph nodes	NSCLC	ADT +/− salvage EBRT	Follow-up (patient refused any treatment)
#4	80	RP (2003)	8.77	2014	Biochemical recurrence	Lymph node	Melanoma	ADT +/− salvage EBRT	Excision of melanoma metastases
#5	71	EBRT (2003) + ADT (stopped)	3.16	2012	Biochemical recurrence	Lung	NSCLC	Salvage EBRT	Lung lobectomy + salvage EBRT
#6	74	RP (2012)	1.4	2012	Persistence of increased PSA after RP	Lung	NSCLC	ADT	Lung lobectomy + salvage EBRT
#7	80	RP (2012) + ADT (stopped)	0.3	2013	Biochemical recurrence	Rectum	Colorectal cancer	ADT +/− salvage EBRT	Anterior resection of rectum + ADT

*ADT* androgen deprivation therapy, *EBRT* external beam radiation therapy, *CHT* chemotherapy, *NSCLC* non small cell lung cancer, *PCa* prostate cancer, *PSA* prostate serum antigen, *Pt* patient, *RP* radical prostatectomy

### Case #1

**Fig. 1:**
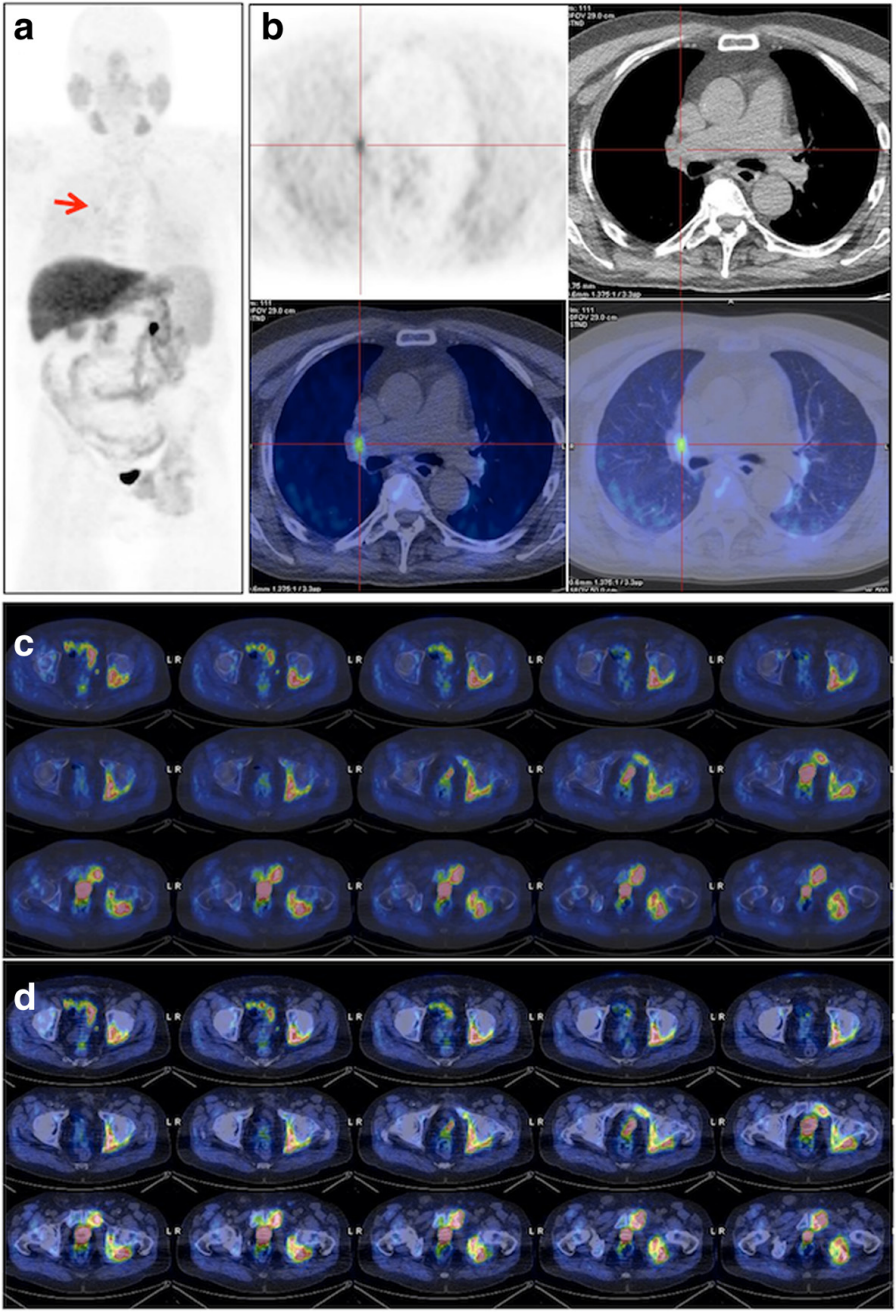
Case#1, 72-yr old man with prostate cancer treated with radical prostatectomy with radiological evidence of bone metastasis. [^18^F]fluorocholine PET/CT shows tracer uptake in the peri-hilar right lung region (**a**, MIP arrow) and in the left side of the pelvis. Transaxial images (**b**) confirm a metabolically active area in a peri-hilar right lung nodule subsequently diagnosed as non small cell lung cancer. Superimposed transaxial PET/CT images (**c**, bone window; **d**, soft tissue window) show [^18^F]fluorocholinee uptake in a vast metastatic lesion involving the left pubis, the left ischium and extending into the adjacent soft tissues. Bone metastases biopsy resulted inconclusive (metastases from adenocarcinoma of unknown origin)

A 72-yr old man affected by PCa was treated by radical prostatectomy (2004) and androgen deprivation therapy (ADT) (ongoing) presented in 2010 PSA raising (0.11 ng/mL) and pain localized at pelvis and left leg. An enhanced computed tomography (CT) scan of the abdomen identified a lesion of the left ischium involving the neighboring soft tissues. In the clinical suspicion of a PCa metastases, a [^18^F]Cho PET/CT was required. PET/CT detected tracer uptake in a peri-hilar right lung nodule (SUV_max_ = 3.9) and in the known left pelvic lesion (SUV_max_ = 6.6) (Fig. 1). The biopsy of the right lung nodule diagnosed a non small cell lung cancer (NSCLC), while that of the bone metastases was inconclusive (metastases from adenocarcinoma of unknown origin resulting positive for both cheratin and AE1/AE3 and negative for both TTF1 and PSA). Based on histological findings the patient started platinum-based chemotherapy plus palliative external beam radiation therapy (EBRT) for the bone metastases.

### Case #2

**Fig. 2:**
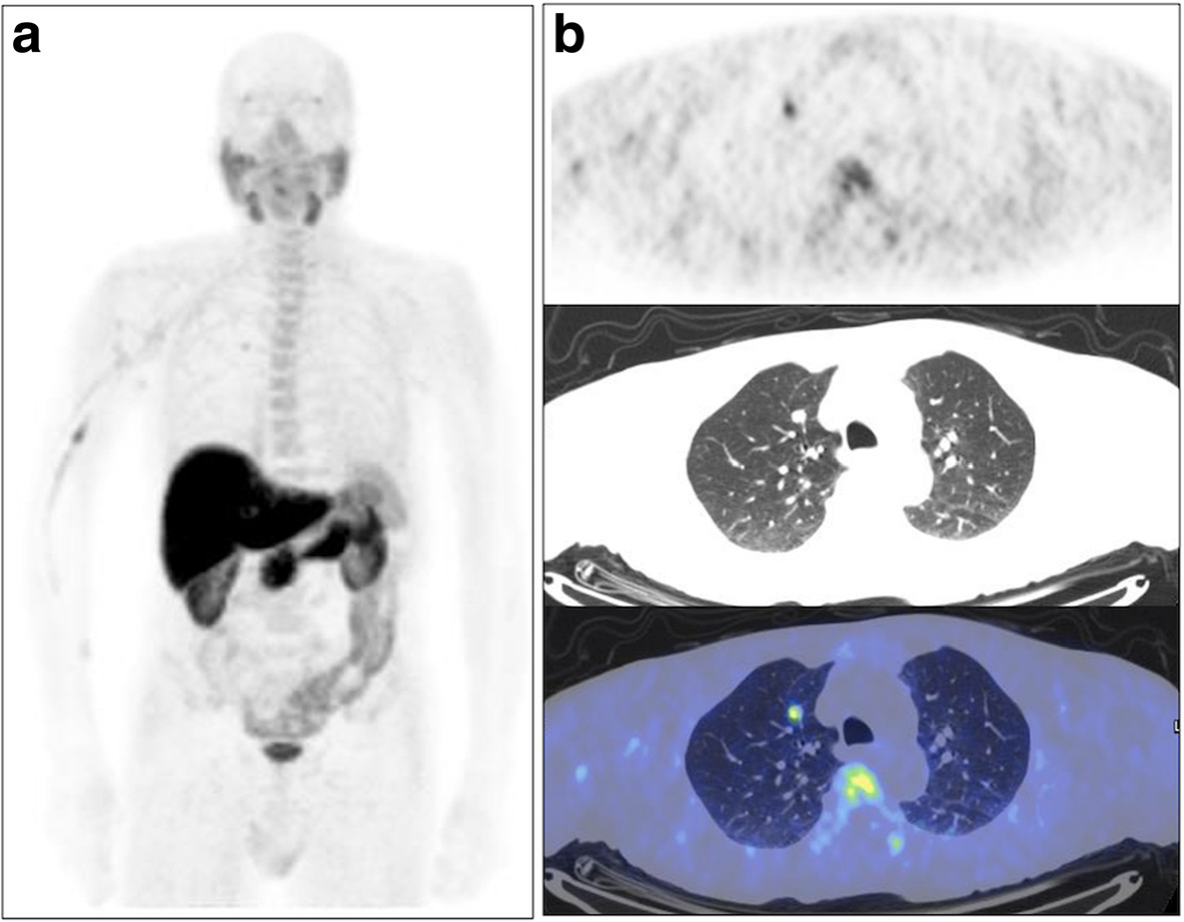
Case#2, 71-yr old man presenting raising PSA values after radical surgery for prostate cancer. The patient was also surgically treated for lung cancer. MIP (**a**) and axial (**b**) [^18^F]fluorocholine PET/CT images show an area of focal radiopharmaceutical uptake in the right lung, subsequently proven to be non small cell lung cancer

A 71-yr old man was treated with radical prostatectomy (1998) for PCa and subsequently with salvage EBRT (2009) for local recurrence. In 2010 he underwent a right lung lobectomy for a NSCLC (pT1N0M0). After salvage EBRT, PSA value although decrease, was not void (0.08 ng/mL) and a restaging [^18^F]Cho PET/CT was performed. PET/CT identified an area of [^18^F]Cho uptake in a small nodule of the right lung (SUV_max_ = 2.7, Fig. 2). Biopsy was performed. The final diagnosis was consistent with NSCLC recurrence and a platinum-based chemotherapy was started.

### Case #3

**Fig. 3:**
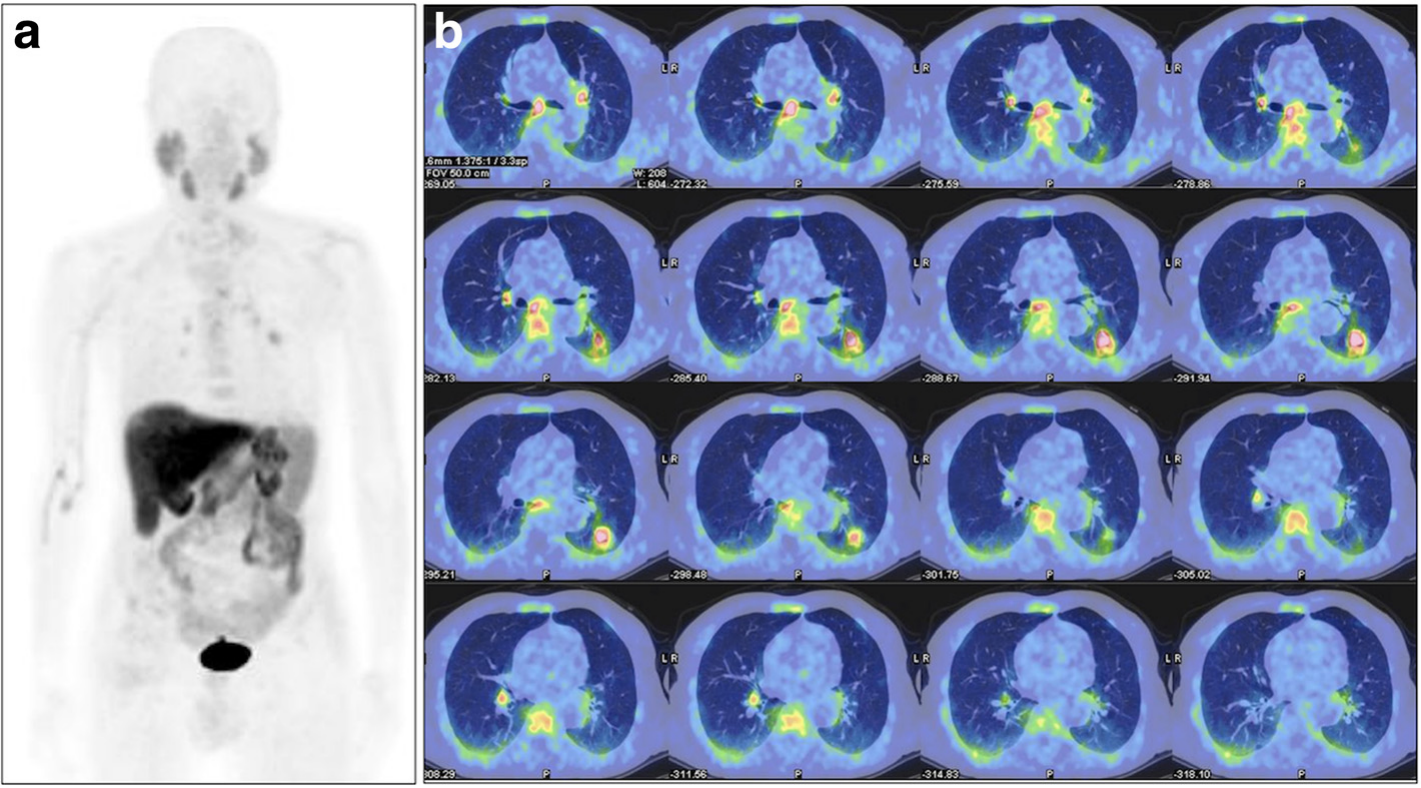
Case#3, 84-yr old man treated with radiation therapy for prostate cancer and with surgery for a basocellular cancer of the skin, presenting with increased PSA values. MIP (**a**) and fused [^18^F]fluorocholine PET/CT axial (**b**) images show increased radiopharmaceutical uptake in the left lung and in multiple hilar and mediastinal lymph nodes subsequently diagnosed as non small cell lung cancer with lymph nodes metastases

A 84-yr old man was treated with EBRT with curative intent (1996) for PCa and with surgery for a basocellular cancer of the skin (2001). Recently PSA values increased up to 2.62 ng/mL. He was symptomless. In order to chose the best treatment option (ADT +/− salvage EBRT) a [^18^F]Cho PET/CT was required. [^18^F]Cho PET/CT identified tracer uptake in a left lung nodule (SUV_max_ = 5.1) and in some hilar-mediastinal lymph nodes (SUV_max_ = 3.3-4) as shown by Fig. 3. PET/CT findings were confirmed by histology which diagnosed a NSCLC with lymph nodes metastases. Despite the diagnosis, the patient based on his good clinical condition and the age, refused any type of treatment for NSCLC, including surgery and chemotherapy. Any treatment for PCa has not been taken avoiding possibly side effects since the prognosis of NSCLC with lymph nodes metastases, was considered the driver for the therapeutic decision.

### Case #4

**Fig. 4:**
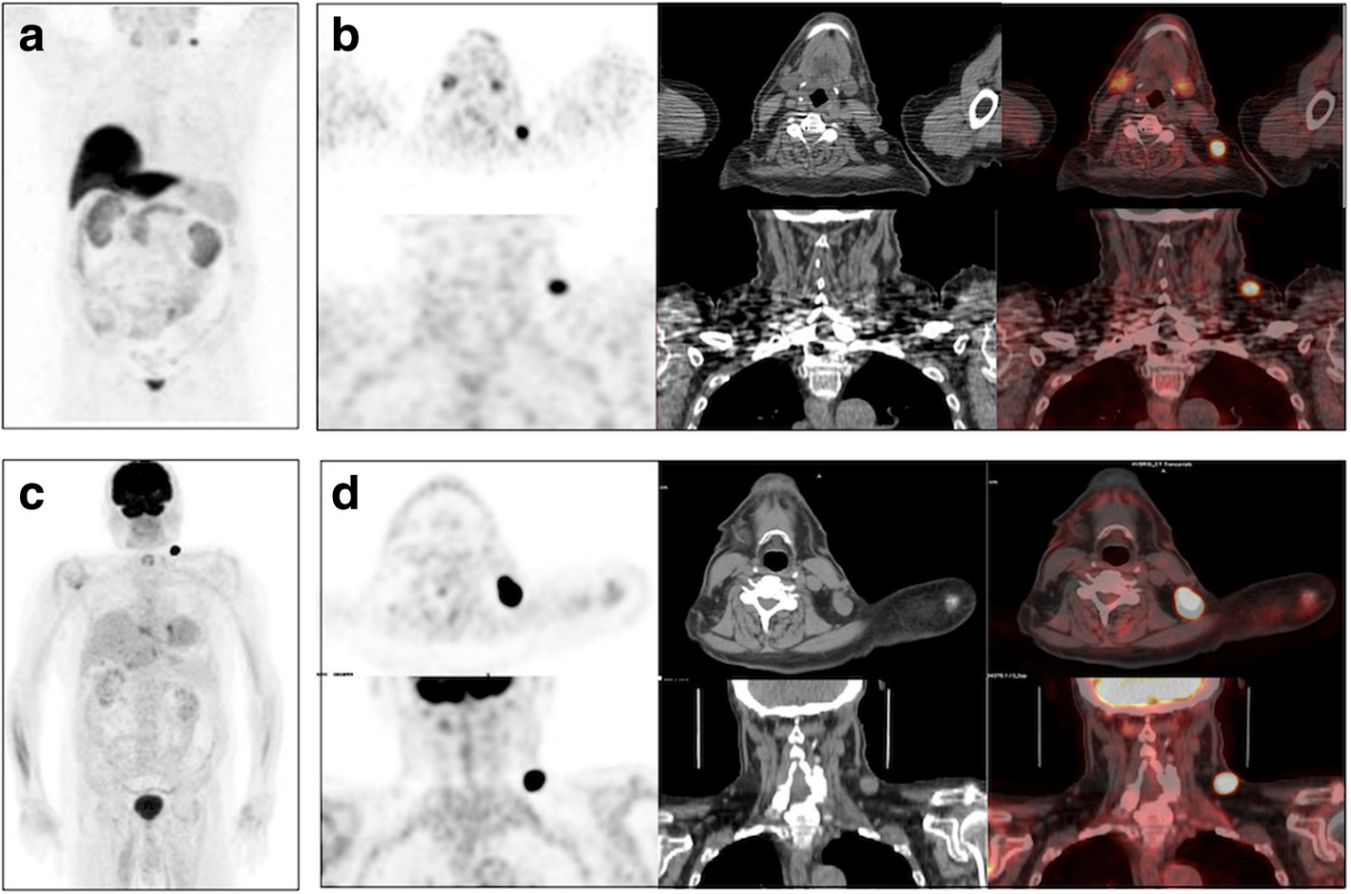
Case #4, 80-yr old man treated with radical prostatectomy for prostate cancer, presenting with progressive PSA raise. MIP (**a**), axial and coronal (**b**) [^18^F]fluorocholine PET/CT images show an area of intense [^18^F]fluorocholine uptake in a left supraclavicular lymph node. The patient underwent a [^18^F]FDG-PET/CT, that confirms the finding as shown by MIP images (**c**), transaxial and coronal images (**d**). Biopsy diagnosed a nodal metastases from melanoma

A 80-yr old man was treated with radical prostatectomy (2003) for PCa and more than 10 years later PSA increased up to 8.77 ng/mL. He was symptomless and in good clinical condition. In order to chose the best treatment option (ADT +/− salvage EBRT) a [^18^F]Cho PET/CT was required. PET/CT identified the presence of an area of [^18^F]Cho uptake in a left supraclavicular lymphadenopathy (SUV_max_ = 15). Patient was also scanned with [^18^F]FDG-PET/CT which confirmed the finding (SUV_max_ = 43; Fig. 4). Subsequently was performed an excisional biopsy which diagnosed melanoma metastases. The patient had no melanoma’s history. Hence he underwent clinical examination and he repeated a [^18^F]FDG-PET/CT in order to diagnose the primary site of the melanoma which resulted both negative. Considering the prognosis quod vitam more linked to melanoma than PCa, it was not taken into account any therapy for PCa and the patients was candidate only to follow-up.

### Case #5

A 71-yr old man with PCa treated by EBRT with curative intent (2003) and ADT (stopped in 2008) presented in 2012 increase in PSA value (3.16 ng/mL). The PET/CT performed for the identification of the site of disease relapse during biochemical recurrence, revealed an area of [^18^F]Cho uptake in a left lung nodule. The biopsy diagnosed a NSCLC. Based on PET/CT findings the patient was treated by left lobectomy for NSCLC. Salvage EBRT on prostatic bed was also performed.

### Case #6

A 74-yr old man was treated with radical prostatectomy (2014) for PCa. However, PSA remained high after surgery reaching a nadir of 1.4 ng/mL. In order to restage the disease and chose the best treatment option, a [^18^F]Cho PET/CT was performed. PET/CT identified the presence of an area of [^18^F]Cho uptake in a right lung nodule subsequently diagnosed as NSCLC. Hence he underwent lung lobectomy plus salvage EBRT on prostatic bed and follow-up without any evidence of recurrence for both prostate and lung cancers.

### Case #7

**Fig. 5:**
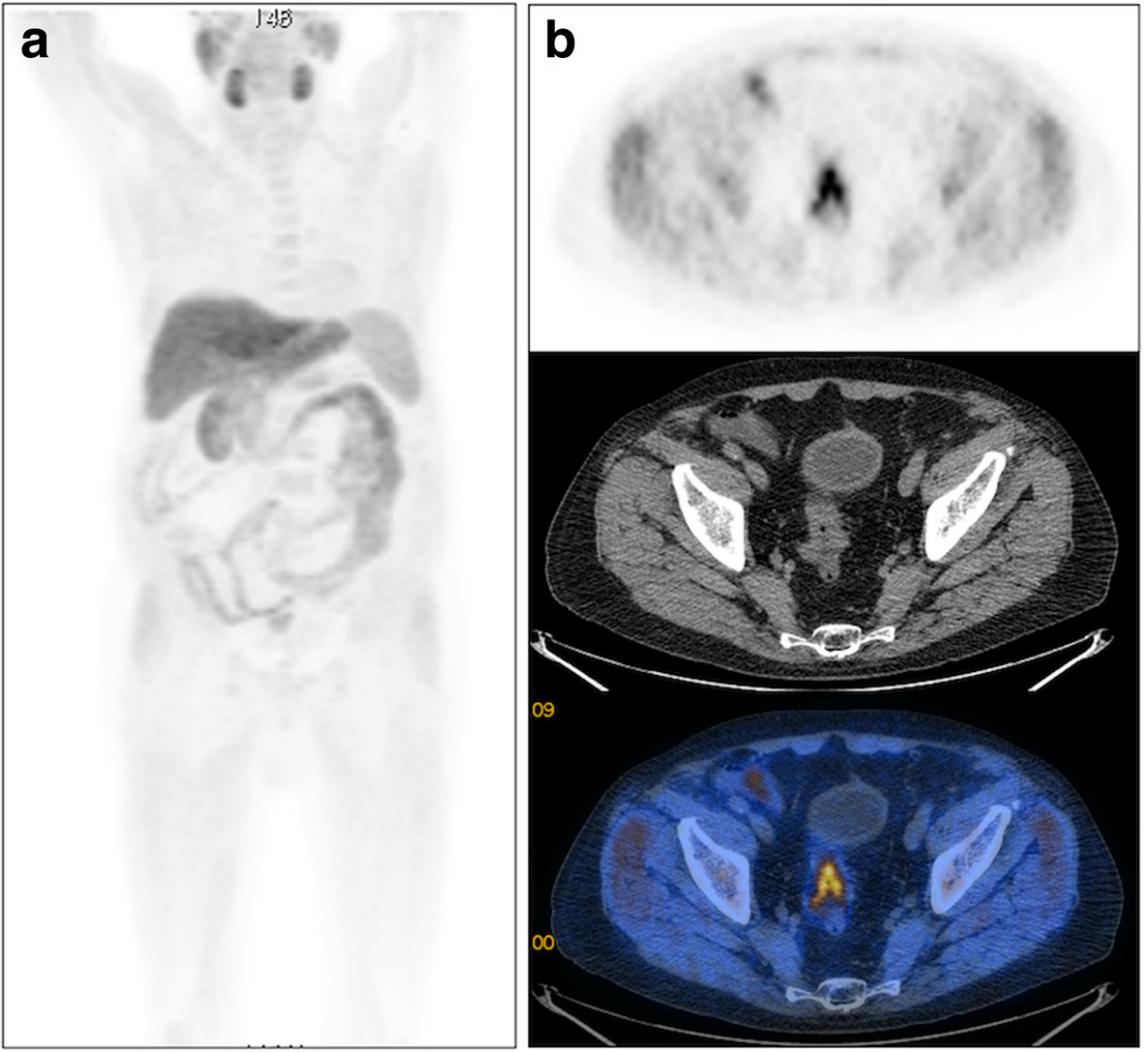
Case#7, [^18^F]fluorocholine PET/CT in a 80-yr old man treated with increased PSA after radical prostatectomy for prostate cancer. MIP (**a**) and axial (**b**) image show a metabolically active area in the rectum which was subsequently diagnosed as colorectal adenocarcinoma (pT3N0)

A 80-yr old man was treated with radical prostatectomy in November 2012 for PCa. In 2013 PSA started to increase up to 0.3 ng/mL. In order to chose the best treatment option (ADT +/− salvage EBRT) the patient underwent a [^18^F]Cho PET/CT which identified the presence of a “hot spot” of [^18^F]Cho in the rectum (SUV_max_ = 7.6, Fig. 5) which was subsequently diagnosed as colorectal cancer. The patient was surgically treated with anterior resection of rectum and then he started ADT for PCa recurrence.

## Discussion

Our results confirm the ability of [^18^F]Cho PET/CT to identify tumor lesion(s) other than PCa. Nevertheless, in PCa patients it may be difficult to distinguish between PCa metastasis and other malignancies. The possibility of “pitfalls” in the interpretation of radiolabeled choline PET/CT imaging in PCa has been reported by Calabria et al. [11] who demonstrated that a careful evaluation of the CT component of images associated to a complete clinical and instrumental data analysis, and eventually to histology, should be always performed to establish a correct diagnosis.

In our series, focal choline uptake was found in all cases, however the site and the characteristics of the uptake were not considered “usual” for PCa metastases resulting more suggestive for lesion of other origin with the exception of the patient (#1) who had bone metastases. In this specific case, histology concluded for a bone metastases from adenocarcinoma but it wasn’t able to discriminate its origin (NSCLC versus PCa). Anyway in this specific case NSCLC diagnosis has driven the therapeutic decision since its prognosis is worst than PCa. In the majority of our cases, the Cho uptake was localized in the lung. These findings may be explained by looking of incidence of cancers. According to International Agency for Research on Cancer [15] the five most frequent cancers in men in Italy are PCa (23.2 %), lung (14 %), colorectal (13.8 %), bladder (7.6 %), and kidney (4 %). Differently from what was reported by Calabria et al. [11] final diagnosis in all our cases was consistent with NSCLC. Anyway, patients included in this series, had not a known lung nodule in their medical history and the [^18^F]Cho uptake registered in the lung nodule was higher than that reported by Calabria et al. (mean SUV_max_ = 3.9 versus 2.2) [11]. Similarly the [^18^F]Cho uptake in lymph nodes was not due to inflammation, but it was related to NSCLC (#3) and melanoma (#4) metastases, respectively. In literature we did not find any case of melanoma previously imaged by [^18^F]Cho PET/CT. Noteworthy in this specific case, PSA was relatively high (8.77 ng/mL) and the hypothesis of a nodal metastases from PCa was realistic despite the site (supraclavicular) was considered unusual and therefore was decided to go on with biopsy. As recently pointed out by How Kit et al. [16], doubtful lesions should be always biopsied. They reported a large series of patients with rising PSA and known or suspected second malignancy imaged by [^18^F]FDG and [^18^F]Cho PET/CT. Anyway, they found that [^18^F]Cho/[^18^F]FDG SUV_mean_ ratio cannot differentiate PCa recurrences from other cancer types concluding that when both diagnoses are suspected, lesions should be biopsied. In our series, only one patient was imaged using both [^18^F]FDG and [^18^F]Cho PET/CT. The availability of this information for only one patient, precluded any further comment. Moreover, PET/CT images were acquired using different scanners, affecting comparability of SUV_max_. However, SUV_max_ was reported to provide trough a simple index, the degree of tracer uptake as well as to compare our findings to literature data, but it was not used for diagnostic purpose.

Differently from that reported by Garcia et al. [14], in all our cases [^18^F]Cho PET/CT resulted positive, impacting on patients’ management, although any PCa recurrence wasn’t identified.

The detection rate of choline PET, reported in literature in the restaging phase, varies between 21 and 82 %, mainly depending on the site of recurrence and PSA levels (detection rate >50 % in case of a median PSA level of 2 ng/ml; detection rate <30 % in case of a PSA level of < 1 ng/ml) [17]. In the setting of PSA relapse after local therapy different treatment strategies can be chosen either salvage EBRT alone or in combination with anti-androgen therapy or anti-androgen therapy alone [18]. The latter option is applied in a palliative intent and should be considered in patients with bad performance status, patients with high PSA values (>2 ng/mL) with a high risk of metastatic disease or in patients where salvage radiotherapy cannot be carried due to other reasons (e.g., previous pelvic EBRT) [19]. In this series of patients, the biochemical PCa recurrence was manage according to the standard of care (EBRT or ADT) with some exceptions. Nonetheless, in all patients the therapeutic approach was modified or implemented based on PET/CT results. In the patient #1 who was candidate to chemotherapy, the regimen adopted was completely different after the diagnosis of NSCLC compared to what would had been used in the case of PCa metastases (platinum-based versus docetaxel) [20, 21]. In the patient #2 clinicians decided for a watch and wait, postponing the initiation of the ADT due to the relative low PSA level and the negativity of [^18^F]Cho PET/CT for PCa recurrence. The patient #3 refused any treatment, while in the patient #4 the prognosis of melanoma drove the therapeutic decision. In all other cases, the change of management included chemotherapy or surgery (+/− EBRT) instead of ADT and/or salvage EBRT alone.

**Fig. 6:**
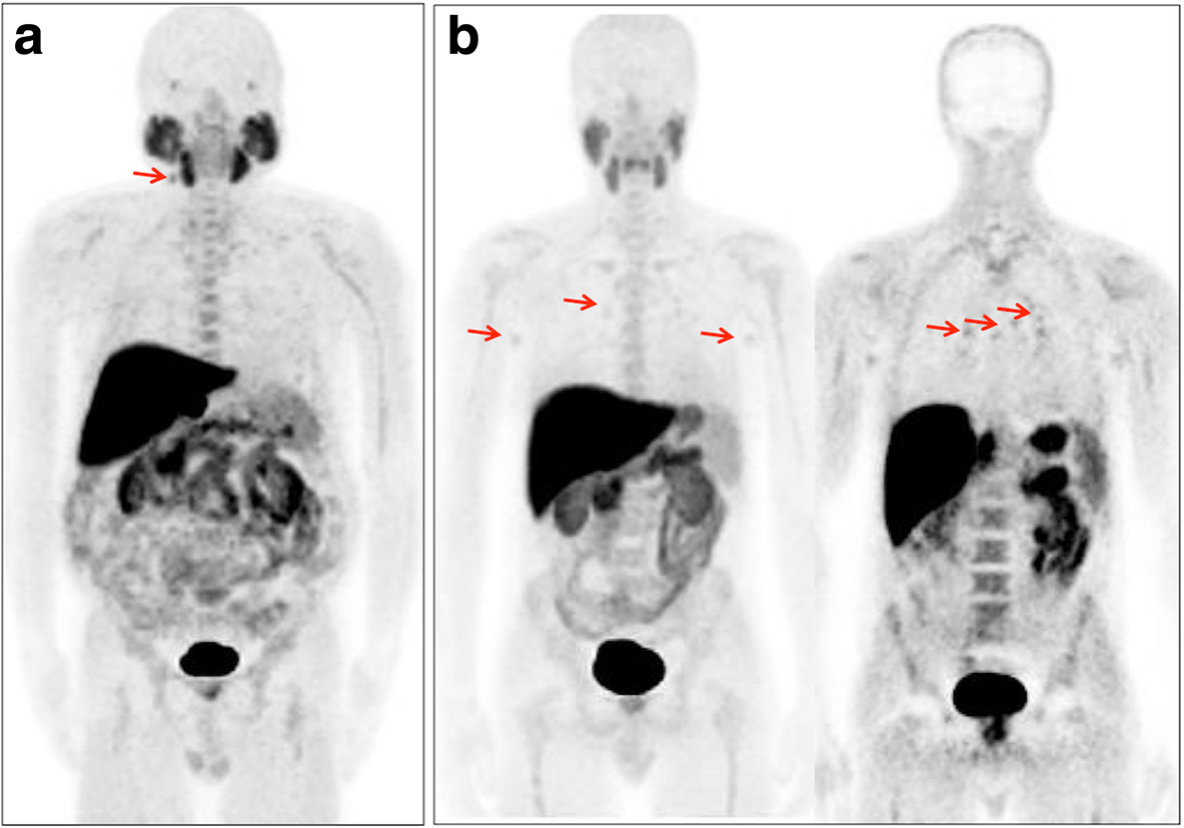
Examples of pitfalls at [^18^F]fluorocholine PET/CT in presence of inflammation. Mild [^18^F]fluorocholine uptake might be present in reactive lymph nodes as shown in these patients (not included in the present series but reported as examples) who present along with prostate lodge recurrence from prostate cancer increased [^18^F]fluorocholine uptake in reactive cervical lymph node (**a**, MIP arrow) and bilateral axillary and hilar/mediastinal lymph nodes (**b**, MIP and PET coronal arrows)

Anyway, as previously pointed up [11, 22, 23], reading a radiolabeled Cho PET/CT, it should take into account the pharmacokinetic and the biodistribution of the radiopharmaceutical as well as the possibility of pitfalls (e.g. parathyroid or thyroid adenomas [24, 25], bilateral hilar-mediastinal lymph nodes [11], Fig. 6) since if it is true that the diagnosis of a second malignancy impacts on patients’ management [14], it is more true that unnecessary procedures and examinations should be avoided in cancer patients unless a very high suspicion.

## Conclusion

To conclude, as previously reported our findings are consistent with the possibility of choline uptake in malignant lesion(s) other than PCa metastases and confirm the needed to accurately ensure any possible “unusual” site of choline uptake of the tracer by histology in order to exclude the presence of a concomitant malignancy.

## Abbreviations

[^18^F]Cho: Fluorocholine
ADT: Adrogen deprivation therapy
Cho: Choline
CT: Computed tomography
EBRT: External beam radiotherapy
NSCLC: Non small cell lung cancer
PCa: Prostate cancer
PET: Positron emision tomography
PET/CT: Positron emissione tomography/computed tomography
PSA: Prostate specific antigen
SUV_max_: Maximum standardized uptake value
